# An automated in vitro model for the evaluation of ultrasound modalities measuring myocardial deformation

**DOI:** 10.1186/1476-7120-8-40

**Published:** 2010-09-07

**Authors:** Albin Stigö, Peter Johansen, Morten Ø Jensen, Kim Sivesgaard, Hans Nygaard, Erik Sloth

**Affiliations:** 1 Department of Anesthesia and Intensive Care, Aarhus University Hospital, Skejby, Aarhus, Denmark; 2 Department of Cardiothoracic and Vascular Research, Aarhus University Hospital, Skejby, Aarhus, Denmark; 3 Institute of Clinical Medicine, Aarhus University Hospital, Skejby, Aarhus, Denmark

## Abstract

**Background:**

Echocardiography is the method of choice when one wishes to examine myocardial function. Qualitative assessment of the 2D grey scale images obtained is subjective, and objective methods are required. Speckle Tracking Ultrasound is an emerging technology, offering an objective mean of quantifying left ventricular wall motion. However, before a new ultrasound technology can be adopted in the clinic, accuracy and reproducibility needs to be investigated.

**Aim:**

It was hypothesized that the collection of ultrasound sample data from an in vitro model could be automated. The aim was to optimize an in vitro model to allow for efficient collection of sample data.

**Material & Methods:**

A tissue-mimicking phantom was made from water, gelatin powder, psyllium fibers and a preservative. Sonomicrometry crystals were molded into the phantom. The solid phantom was mounted in a stable stand and cyclically compressed. Peak strain was then measured by Speckle Tracking Ultrasound and sonomicrometry.

**Results:**

We succeeded in automating the acquisition and analysis of sample data. Sample data was collected at a rate of 200 measurement pairs in 30 minutes. We found good agreement between Speckle Tracking Ultrasound and sonomicrometry in the in vitro model. Best agreement was 0.83 ± 0.70%. Worst agreement was -1.13 ± 6.46%.

**Conclusions:**

It has been shown possible to automate a model that can be used for evaluating the in vitro accuracy and precision of ultrasound modalities measuring deformation. Sonomicrometry and Speckle Tracking Ultrasound had acceptable agreement.

## Background

Accurate assessment of myocardial function is of paramount importance for the evaluation of the ill patient. In the past, the assessment of cardiac function has been based on qualitative or when best, semi quantitative methods, which are highly observer dependent [1,2]. Speckle Tracking Ultrasound (STU) has recently been introduced in the clinic and is based on frame-to-frame tracking of characteristic ultrasound speckle patterns within the myocardium [3]. From this information, strain and strain rate can be derived. In contrast to Tissue Velocity Imaging [3-5] (TVI), STU is angle independent [4,6].

STU has been adopted in the clinic without much data to support its accuracy and reproducibility. Ideally, before a new ultrasound technology is used in human or animal studies it should have been investigated for accuracy and reproducibility in a fully controllable environment, to disclose any fundamental limitations of the method.

Typically such studies are carried out in human subjects or animal models [3] with an invasive or noninvasive reference or in an *in vitro *setup [7,8]. An in vitro setup has the advantage of being fully controllable, and the influence of parameters such as strain and strain rate can be investigated systematically.

In a previous study we described a dynamic ultrasound phantom and showed that STU was independent of insonation angle and gain [6]. Such an evaluation implies acquiring a large amount of data and is therefore very time consuming. A model where data acquisition and analysis are automated is a much wanted tool. Furthermore, such a model would also, in a reproducible way, enable comparison of equipment from different manufactures.

The frame rate vs. spatial resolution relationship in ultrasound imaging is complex [9]. At a fixed image size a higher frame rate can only be achieved by compromising image resolution. It is of interest to study how this relationship affects the precision of STU. Since STU uses a pixel tracking algorithm [7], a higher resolution would theoretically allow for greater spatial precision (resolution) while a higher frame rate would allow for greater temporal precision.

## Materials and methods

### The ultrasound tissue-mimicking phantom

The ultrasound tissue-mimicking phantom has previously been described [6]. It consists of water, gelatin, psyllium fibers and a preservative mixed, and cast in a cylindrical mold.

The water and gelatin form a solid gel. The psyllium fibers provides a ultrasound scattering pattern similar to the myocardium.

The speed of sound and acoustic impedance in the phantom was measured to 1530 m/s and 1.53 kg/m^2^s, respectively. The average speed of sound and acoustic impedance in soft tissue is 1.54 m/s and 1.58 kg/m^2^s. The phantom was kept cool between experiments in order to keep it structurally intact, as gelatin forms a thermally reversible gel.

### Sonomicrometry

**Figure 1:**
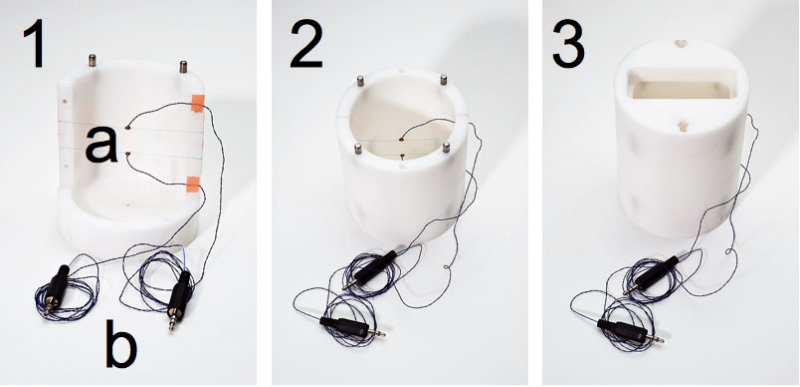
**Tissue-mimicking phantom mold**. The tissue-mimicking phantom was cast in a mold (1, 2 and 3). This consisted of a lid, bottom, and two sides which were held together by metal pegs. Sonomicrometry crystals were suspended on sutures in the mold, before the liquid phantom mass was added. 1) Bottom and one side. a) Crystals on sutures. b) Connectors 2) Bottom and two sides. 3) Assembled.

Sonomicrometry (Sonometrics, London, Ontario, Canada) was used as a reference measure of strain in the phantom. Sonomicrometry (Sono) is an ultrasound ranging technique using small embedded piezoelectric crystals. Two crystals were embedded in the phantom, during the casting they were suspended on thin sutures (6-0), figure 1. With the phantom in its solid state the crystals were held in place by the surrounding material and the sutures no longer had any function.

The crystals were connected to a transceiver controlled by a workstation running SonoLAB software (Sonometrics). The time of flight was measured by a 100 MHz counter, giving a spatial resolution of 24 *μ*m (Sonometrics). The distance between the crystals was sampled at 200 Hz in both directions (from crystal 1 to 2 and 2 to 1).

### Vibration exciter

**Figure 2:**
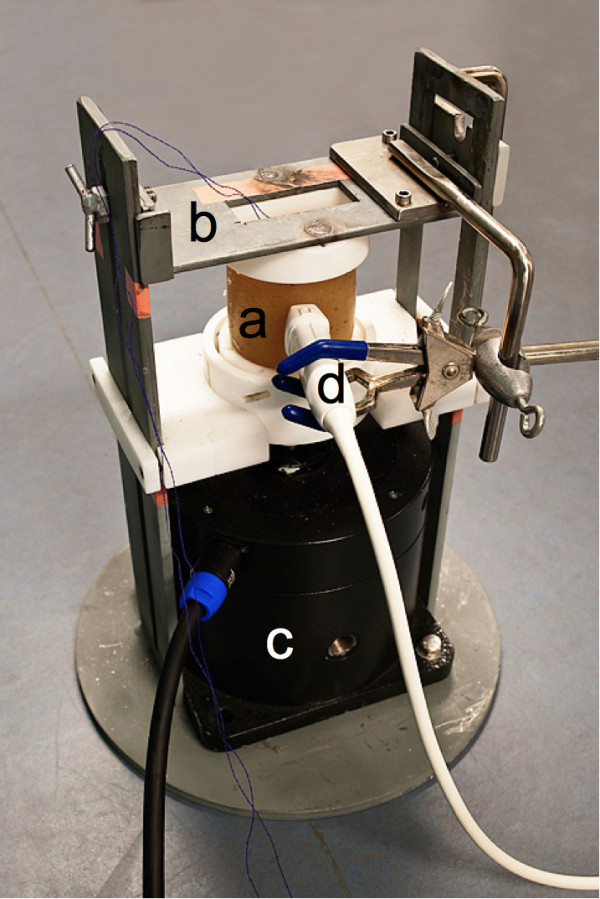
**Stable stand**. The tissue-mimicking phantom (a) was mounted in a stable stand (b) between the top (lid) and bottom of the mold in which it was cast (after removing the two sides). The lid was fixed to a horizontal bar by two metal pegs (b). The bottom was connected to a variable exciter (c) through a linking device. An ultrasound probe (d) was held in place by an adjustable clamp and good acoustic contact was assured by applying ultrasound gel at the contact area.

A vibration exciter (type 4808, Brüel and Kjær, Nærum, Denmark) was used for dynamically compressing the phantom, figure 2. The exciter has linear properties and is able to simulate any physiological or pathological strain curves with peak strains $\left(\frac{\Delta L}{L}\right)$ from 0 to 20%.

### Mounting stand

The phantom was placed in a stable mounting stand and held in place by the top (lid) and bottom parts of the mold, figure 2. The top part of the mold was fixed to a horizontal bar. A hole was cut in the top of the bar to allow scanning in the axial direction. The bottom part of the mold was connected to the vibration exciter's actuator and served as a compression plate.

### Waveform generation

The compression of the phantom was controlled by a PC workstation running custom software. A digital waveform was generated in software and converted to an analog voltage by a DAQmx (PCI-6110) data acquisition interface (National Instruments, Austin, TX, USA). This signal was connected to an amplifier (type 2719, Brüel and Kjær, Nærum, Denmark) that powered the vibration exciter.

### Ultrasound scanner

The ultrasound system used was a Vivid E9 (GE Healthcare, Horten, Norway) equipped with a M5 S cardiac probe. All settings apart from frame rate (50, 60 and 86 frames/second) were constant through out the experiments: Gain -6dB, depth 9 cm, focus depth 4.5 cm and sector width 75°. Second harmonic imaging with a fundamental frequency of 1.7 MHz was used.

### Remote control

**Figure 3:**
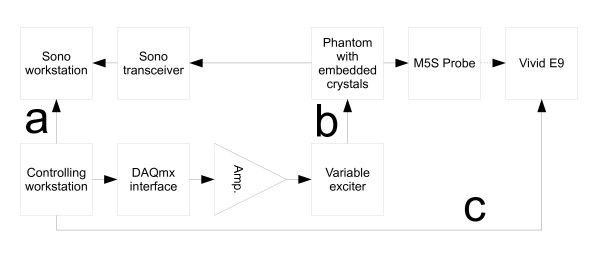
**Experimental setup**. Schematic diagram of the experimental setup. The sonomicrometry workstation was remotely triggered by a link (a) connected to the controlling workstation. The tissue-mimicking phantom was connected to the variable exciter through a linking device (b). The Vivid E9 ultrasound scanner was remotely controlled (c) by the controlling workstation.

All parts of the experimental setup were controlled by a single workstation, figure 3. Remote control links were set up to control the ultrasound scanner and the Sono workstation.

The Sono workstation had a TTL-level input, which could be configured in software to trigger the save function.

In pilot studies we used the Vivid 7 (GE Healthcare) scanner. This scanner has a remote control interface in the form of a foot switch port. Electronically controlled switches were connected to this port. In our experiment we used the newer Vivid E9 scanner. This scanner did not have a remote control port and a custom interface consisting of servo motors and a rack that could be fixed to the scanner was designed. Servo motors mounted on the ultrasound scanner would then push the buttons. Due to interfering frequency bands for echocardiography and Sono, a method of switching between the two systems was necessary. This was accomplished by gating the transmit pulse from the Sono workstation to the Sono transceiver with a control signal from the controlling workstation, and by freezing the ultrasound scanner while Sono was operating.

**Figure 4:**
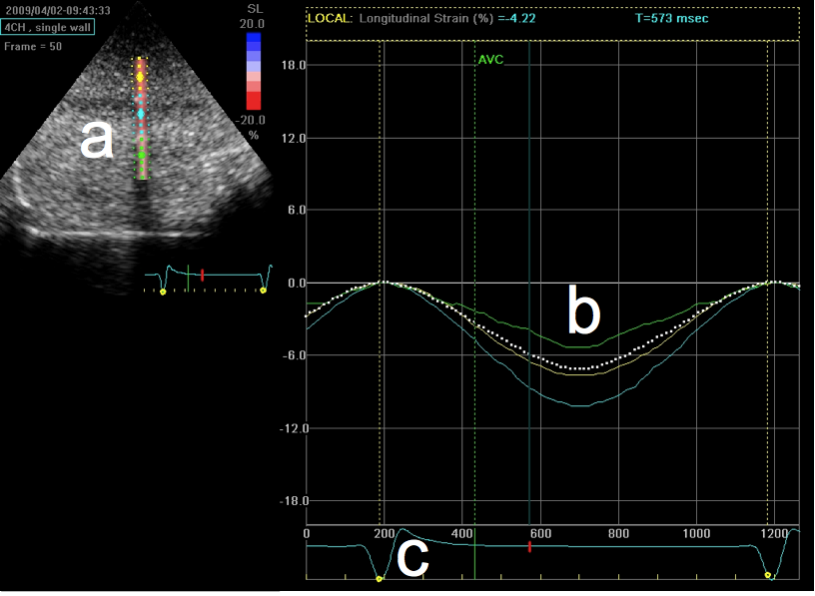
**EchoPAC screenshot**. Data was analyzed in EchoPAC (screenshot). A region of interest (a) was selected and the software analyzed strain in that region (b). A synthesized ECG signal (c) was used to correctly align the strain curves. Strain data was then exported from this view to a spreadsheet for further analysis.

A square wave with 5% duty cycle, in sync with the compression cycle, was generated by the software and connected to the ultrasound scanner's ECG input through a voltage divider network. This facilitated quicker offline analysis, see figure 4.

### Custom software

Custom software was developed for controlling the experimental setup and for analyzing data. All custom software was developed in the Python programming language (Python Software Foundation, Hampton, NH, USA).

### Calibration methodology

Calibration of the setup was accomplished with the phantom mounted in the stable stand. A sine wave was generated and gain was adjusted to be within the working limits of the exciter. A 5% preload was applied to the phantom to ensure contact between the phantom and the compression plate throughout the entire compression cycle.

### Experimental conditions

The setup was used to evaluate the precision of STU.

After mounting the phantom in the stable stand the M5S probe was positioned in the bracket and good acoustic contact was assured. Ultrasound gel was applied at the contact area between the probe and the phantom.

The controlling workstation was programmed to acquire 50 dynamic images for each of the four studied displacement frequencies (1, 2, 4 and 8 Hz). A sine waveform was used for compressing the phantom. The amplitude was randomly set to one of 10 different voltage levels roughly corresponding to peak strain levels between 5 and 20% (depending on precise calibration). This was repeated for each studied frame rate (50, 60 and 86 frames/second) and for each angle (0° and 90°), yielding 1200 measurement pairs. All experiments were conducted at a room temperature of 22°C.

### Data analysis

The 2D ultrasound data was exported to an EchoPAC workstation (GE Healthcare, Horten, Norway) for off-line STU analysis [10]. The method for assessing peak strain in the phantom using STU-analysis has previously been described in detail [6].

Strain curves were exported to a workstation running custom software that was used to extract peak strain. Sono data was exported to a workstation for analysis. The average between the two sampled distances (from crystal 1 to 2 and 2 to 1) was used, and data was filtered using a moving median filter (kernel size 3) to remove outliers caused by electrical noise. To ensure that the applied filtering was effective Sono strain curves were then visually inspected.

Peak strain from STU 2D strain analysis and Sono were combined and exported to a spreadsheet for overview. Individual results were displayed in scatter and mean difference plots [11].

## Results

We automated the acquisition and analysis of sample data. Six sets of test data was collected with 200 measurement pairs in each set. Data was collected at 200 measurement pairs in 30 minutes (compared to 80 pairs in 30 minutes using the old setup).

**Figure 5:**
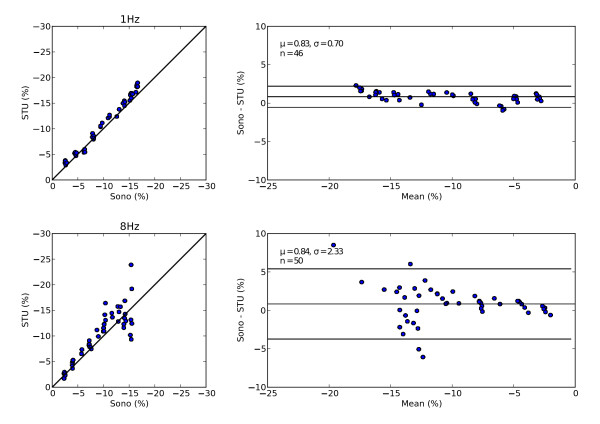
**Example plots**. Peak strain in the tissue-mimicking phantom was measured using both sonomicrometry and Speckle Tracking Ultrasound. Agreement between the two methods were visualized in scatter and mean difference plots. Top: Scanning angle: 0°, frame rate: 50 Hz, compression frequency 1 Hz. Bottom: Scanning angle: 0°, frame rate: 50 Hz, compression frequency 8 Hz.

The collected sample data was analyzed and agreement between STU and Sono was calculated. Agreement between STU and Sono for each combination of frame rate, insonation angle and compression frequency is presented in table 1. Typical plots are shown in figure 5. During analysis 25 measurements were excluded because of the EchoPAC software failing to analyze them. We found good agreement between STU and Sono.

**Table 1:** Agreement between Sono and STU.

Frame rate (Hz)	Insonation angle (°)	Compression freq. (Hz)	Agreement (%)	n
50	0	1	0.83 ± 0.70	46
		
		2	1.40 ± 0.96	50
		
		4	1.63 ± 1.08	50
		
		8	0.84 ± 2.33	50
	
	90	1	0.14 ± 2.54	50
		
		2	-0.18 ± 1.68	50
		
		4	0.27 ± 1.76	50
		
		8	0.64 ± 3.90	50

60	0	1	1.40 ± 2.78	49
		
		2	1.08 ± 2.02	50
		
		4	0.83 ± 1.84	50
		
		8	-1.13 ± 6.46	50
	
	90	1	-1.48 ± 1.66	44
		
		2	-0.92 ± 1.88	50
		
		4	-0.05 ± 2.02	50
		
		8	0.28 ± 2.78	50

86	0	1	-0.70 ± 3.10	46
		
		2	-0.87 ± 1.38	50
		
		4	-0.29 ± 1.26	50
		
		8	-0.60 ± 0.71	50
	
	90	1	-2.99 ± 2.24	40
		
		2	-1.88 ± 1.46	50
		
		4	-0.93 ± 0.82	50
		
		8	-0.35 ± 1.78	50

n is number of analyzed measurement pairs.

## Discussion

It can be seen from the results that a higher frame rate was necessary to accurately assess peak strain at higher deformation frequencies during larger deformations. In our experiments we used 8 Hz as the highest deformation frequency. Even though 8 Hz (480 bpm) is not a physiological heart rate this result is interesting from a clinical perspective as it shows that the selection of frame rate is not without influence when assessing rapid movements. If the frame rate is too low, important events might be filtered out. Since frame rate is dependent on sector width and scanning depth, our results support the idea that it is necessary to keep the visualized area as small as possible to achieve the best accuracy.

During analysis 25 measurements were excluded. It is notable that all dynamic images that the EchoPAC software failed to analyze were obtained at a compression frequency of 1 Hz. It seems possible that this was due to a bug in the EchoPAC software.

Since many producers of ultrasound equipment have their own solutions for STU, our model could be used for comparing their performance without bias. It could also be used for periodically testing available equipment making sure it's free from errors and is performing to specifications, something that is often overlooked in the clinic.

Numerous in vitro setups have previously been used to evaluate methods for quantifying myocardial mechanics. They have often relied on an uncertain reference [7,8,12]. As opposed to these models we used Sono which is considered the golden standard when estimating myocardial deformation in animal models [3,13].

In our experiments a simple sine wave was used for compressing the phantom but the variable exciter along with appropriate DSP software is capable of simulating any synthesized or recorded waveform (within the bandwidth of the system). We believe this allows for greater flexibility than previously used methods based on motors [7] or pumps [12].

This study has been able to demonstrate that it is possible to construct a model that can produce reliable data in the initial testing of a new ultrasound technology. The automation of the setup with the use of a central workstation has to our knowledge not been described before. It enables a high rate of data collection, minimizes the risk of human error and ads unprecedented reproducibility.

## Limitations

The in vitro phantom presents an optimal environment for strain measurements with optimal image quality, and a homogenous speckle pattern in the entire region of interest (ROI). The depth of the crystals (4 cm) is also less than the normal depth of the myocardium.

The small size of the phantom led to strong echoes from the walls and some artifacts in the images outside of the phantom. Coating or embedding the phantom in an ultrasound absorbing material could minimize these strong echoes. We did not however experience any problems with obvious artifacts inside the phantom and did not further optimize in this respect.

Because of the large difference in acoustic impedance between the Sono crystals and the phantom material the crystals introduced considerable shadowing. This was not a problem when scanning from a 90° angle but posed a problem when scanning at 0° as the crystals then were in parallel with the beam. This effect was minimized by carefully aligning the beam so that some ultrasound energy was able to pass on the side of the crystal.

As the temperature in the phantom material increased during the experiments, it became softer and would displace more for a given force. Since all our measurements were relative, time to conduct the experiment was short and the rate of change very small, this was not a problem. Also for this reason, precise calibration was not considered crucial.

A way of compensating for the effects of rising temperature and any nonlinearity in the material would be to set up a strain feedback loop from the Sono workstation to the workstation controlling the variable exciter. In this way, the deformation could be held at a constant level and any disturbances compensated for in real-time. This, however, would only be interesting for precise measurements and not necessary for relative strain.

## Conclusions

It has been shown possible to automate the data collection and a large part of the data analysis in an in vitro deformation ultrasound model. Data collection was twice as fast compared to our old model [6]. Analysis of Sonomicrometry data was facilitated by custom software. The developed model was used to test agreement of Speckle Tracking Ultrasound and Sonomicrometry. We found good agreement between STU and Sono in the in vitro model. Best agreement was 0.83 ± 0.70%. Worst agreement was -1.13 ± 6.46%.

## Competing interests

The authors declare that they have no competing interests.

## Authors' contributions

AS optimized the model, wrote the custom software, carried out the preliminary study, analyzed the data and drafted the manuscript. PJ provided technical assistance in development of the model and helped writing the manuscript. MØJ provided technical assistance in development of the model and helped writing the manuscript. KS developed the original version of the model and assisted in writing the manuscript. HN was principal supervisor, determined the acoustic properties of the tissue-mimicking phantom and helped design the preliminary study. ES was project supervisor, conceived the study, participated in its design and coordination and helped drafting the manuscript.

All authors have read and approved the final manuscript.

## References

[B1] Hoffmann R Lethen H Marwick T Arnese M Fioretti P Pingitore A Picano E Buck T Erbel R Flachskampf FA Hanrath P Analysis of interinstitutional observer agreement in interpretation of dobutamine stress echocardiograms Journal of the American College of Cardiology 1996 27 2 330 336 10.1016/0735-1097(95)00483-1 8557902

[B2] McDicken WN Sutherland GR Moran CM Gordon LN Colour Doppler velocity imaging of the myocardium Ultrasound in Medicine & Biology 1992 18 6-7 651 654 10.1016/0301-5629(92)90080-t 1413277

[B3] Amundsen BH Helle-Valle T Edvardsen T Torp H Crosby J Lyseggen E Støylen A Ihlen H Lima JAC Smiseth OA Slørdahl SA Noninvasive myocardial strain measurement by speckle tracking echocardiography: validation against sonomicrometry and tagged magnetic resonance imaging Journal of the American College of Cardiology 2006 47 4 789 793 10.1016/j.jacc.2005.10.040 16487846

[B4] Langeland S Wouters PF Claus P Leather HA Bijnens B Sutherland GR Rademakers FE D'hooge J Experimental assessment of a new research tool for the estimation of two-dimensional myocardial strain Ultrasound in Medicine & Biology 2006 32 10 1509 1513 10.1016/j.ultrasmedbio.2006.06.021 17045871

[B5] Urheim S Edvardsen T Torp H Angelsen B Smiseth OA Myocardial strain by Doppler echocardiography. Validation of a new method to quantify regional myocardial function Circulation 2000 102 10 1158 1164 10973846 10.1161/01.cir.102.10.1158

[B6] Sivesgaard K Christensen SD Nygaard H Hasenkam JM Sloth E Speckle tracking ultrasound is independent of insonation angle and gain: an in vitro investigation of agreement with sonomicrometry Journal of the American Society of Echocardiography: Official Publication of the American Society of Echocardiography 2009 22 7 852 858 19515531 10.1016/j.echo.2009.04.028

[B7] Ashraf M Li XK Young MT Jensen AJ Pemberton J Hui L Lysyansky P Friedman Z Park B Sahn DJ Delineation of cardiac twist by a sonographically based 2-dimensional strain analysis method: an in vitro validation study Journal of Ultrasound in Medicine: Official Journal of the American Institute of Ultrasound in Medicine 2006 25 9 1193 1198 16929021 10.7863/jum.2006.25.9.1193

[B8] Belohlavek M Bartleson VB Zobitz ME Real-time strain rate imaging: validation of peak compression and expansion rates by a tissue-mimicking phantom Echocardiography (Mount Kisco, N.Y.) 2001 18 7 565 571 10.1046/j.1540-8175.2001.00565.x 11737965

[B9] Teske AJ Boeck BWLD Melman PG Sieswerda GT Doevendans PA Cramer MJM Echocardiographic quantification of myocardial function using tissue deformation imaging, a guide to image acquisition and analysis using tissue Doppler and speckle tracking Cardiovascular Ultrasound 2007 5 27 10.1186/1476-7120-5-27 17760964 PMC2000459

[B10] Korinek J Kjaergaard J Sengupta PP Yoshifuku S McMahon EM Cha SS Khandheria BK Belohlavek M High spatial resolution speckle tracking improves accuracy of 2-dimensional strain measurements: an update on a new method in functional echocardiography Journal of the American Society of Echocardiography: Official Publication of the American Society of Echocardiography 2007 20 2 165 170 17275702 10.1016/j.echo.2006.08.031

[B11] Bland JM Altman DG Statistical methods for assessing agreement between two methods of clinical measurement Lancet 1986 1 8476 307 310 2868172

[B12] Hashimoto I Mori Y Rusk RA Davies CH Li X Mack GK Sahn DJ Strain rate imaging: an in vitro "validation" study using a physiologic balloon model mimicking the left ventricle Echocardiography (Mount Kisco, N.Y.) 2002 19 8 669 677 10.1046/j.1540-8175.2002.00669.x 12487636

[B13] Korinek J Vitek J Sengupta PP Romero-Corral A Krishnamoorthy VK McMahon EM Khandheria BK Belohlavek M Does Implantation of Sonomicrometry Crystals Alter Regional Cardiac Muscle Function Journal of the American Society of Echocardiography 2007 20 12 1407 1412 10.1016/j.echo.2007.04.010 17604963

